# Vagal stimulation in heart failure

**DOI:** 10.1007/s00059-021-05076-5

**Published:** 2021-10-30

**Authors:** Veronica Dusi, Gaetano Maria De Ferrari

**Affiliations:** Division of Cardiology, Department of Medical Sciences, Citta della Salute e della Scienza Hospital, University of Turin, Corso Bramante 88, 10126 Turin, Italy

**Keywords:** Neuromodulation, Autonomic regulation therapy, Device therapy, Sympathetic nervous system, Autonomic imbalance, Neuromodulation, Autonome Regulationstherapie, Therapie unter Geräteeinsatz, Sympathisches Nervensystem, Autonomes Ungleichgewicht

## Abstract

Vagal nerve stimulation (VNS) has a strong pathophysiological rationale as a potentially beneficial treatment for heart failure with reduced ejection fraction. Despite several promising preclinical studies and pilot clinical studies, the two large, controlled trials—NECTAR-HF and INOVATE-HF—failed to demonstrate the expected benefit. It is likely that clinical application of VNS in phase III studies was performed before a sufficient degree of understanding of the complex pathophysiology of autonomic electrical modulation had been achieved, therefore leading to an underestimation of its potential benefit. More knowledge on the complex dose–response issue of VNS (i.e., pulse amplitude, frequency, duration and duty cycle) has been gathered since these trials and a new randomized study is currently underway with an adaptive design and a refined approach in an attempt to deliver the proper dose to a more selected group of patients.

## Abstract (de)

Für die Vagusnervstimulation (VNS) als einer sinnvollen Therapie bei Herzinsuffizienz bestehen starke pathophysiologische Argumente. Trotz mehrerer vielversprechender präklinischer und Pilotstudien konnten die beiden großen kontrollierten Studien NECTAR-HF und INOVATE-HF den erwarteten klinischen Nutzen jedoch nicht zeigen. Vermutlich wurde hierbei die klinische Anwendung dieser Therapie durchgeführt, bevor die komplexe Pathophysiologie der autonomen elektrischen Modulation ausreichend verstanden worden war, sodass diese Studien nicht in der Lage waren, das Potenzial der VNS zu demonstrieren. Seit diesen Studien wurde mehr Wissen über die klinische Anwendung der VNS erlangt, insbesondere zur Patientenselektion und zur Art der Stimulation („Dosis“, d. h. Amplitude, Impulsfrequenz [Hz], Dauer der Anwendung und Arbeitszyklus der VNS). Eine neue randomisierte Studie mit einem adaptiven Design wird aktuell durchgeführt, um die Fragen nach der optimalen Dosis der VNS zu beantworten

Autonomic imbalance, essentially consisting in excessive sympathetic activation and concomitant vagal withdrawal, is a pivotal element in the pathophysiology of chronic heart failure with reduced ejection fraction (HFrEF), regardless of the etiology. Accordingly, indirect markers of reduced cardiac vagal output, such as baroreceptor sensitivity (BRS) and heart rate variability (HRV), are associated with a worse hemodynamic profile as well as a lower New York Heart Association (NYHA) functional class [1], and directly convey unfavorable long-term prognostic implications for these patients [2]. Furthermore, episodes of acutely decompensated HF are typically preceded by a further, abrupt inhibition of cardiac vagal activity (in addition to the chronic inhibition; [3]). Unsurprisingly, correction of this autonomic imbalance has become an important therapeutic target in HFrEF. Interventions directly targeting the autonomic nervous system are generally referred to as “neuromodulation” or “autonomic regulation therapy” (ART). This review focuses on electrical vagal nerve stimulation (VNS), which is the most studied device-based ART in the setting of HFrEF [4, 5].

## Vagus nerve anatomy and functions

The vagus nerve (VN) is a mixed nerve, composed of approximately 80% afferent and 20% efferent axonal projections. Efferent fibers originate in the dorsal motor nucleus and in the nucleus ambiguous located in the brainstem, and project to the heart, lungs, larynx, pharynx, stomach, spleen, pancreas, liver, intestines, and ovaries. At the cardiac level, it is now well established that efferent vagal fibers do not directly innervate cardiomyocyte as previously believed, but relay on neurons located in epicardial fat pads, which constitute a complex neuronal network better known as the “intrinsic cardiac nervous system” (ICNS; [6]). The human ICNS contains at least 10 major groupings of ganglionated plexuses, each one providing a preferential, albeit not exclusive, and largely overlapping distribution to different cardiac regions. The right and the left VN do not share exactly the same distribution on the plexuses of the ICNS; as a result, the right VN has a greater influence on the sinus node activity, whereas the left VN has a predominant control over the atrioventricular node function.

From a structural point of view, the VN contains a mixture of different types of nerve fibers, which are organized into bundles (fascicles). Unfortunately, despite decades of extensive research, it is still a matter of debate whether fascicles into the VN are arranged according to the type of fiber (afferent or efferent) they contain or according to their peripheral end-organ distribution (namely, somatotopic arrangement). The available evidence, however, favors the latter possibility [7]. Neuronal fibers within the VN are classified according to their diameter and their conduction velocity, with Aα fibers being the largest and fastest, unmyelinated C fibers the smallest and slowest, and Aβ, Aγ, Aδ, and B fibers intermediate. Cardiac vagal control in mammalians is mediated by B- (efferent) and C‑type fibers (afferent and efferent). Notably, VNS recruits neuronal fibers based on proximity to the stimulation electrode, the local electric field strength, and inversely related to size—with A‑type fibers being recruited first and C‑type fibers last.

## Preclinical data

Vagal nerve stimulation was first studied as an antiarrhythmic intervention. The first report dates back to 1859 [8], when Einbrodt incidentally observed that dogs receiving VNS were less likely to die from direct delivery of electrical current to the ventricles. More than 100 years later, between 1973 and 1978, several studies on anesthetized animals [9–12] confirmed that VNS reduces the risk of ventricular fibrillation (VF) during acute myocardial ischemia. In the study by Myers, the protective effect on VF was not abolished by preventing heart rate (HR) decrease and was only mildly reduced by VN decentralization, therefore suggesting a direct efferent effect of VNS at the ventricular level [12]. A definite mechanistic demonstration came in 1991, when a conscious canine model of sudden death was used to prove that cholinergic antagonism favors VF, while right VNS applied a few seconds after coronary occlusion protects against VF; almost 50% of this protective effect was related to the significant HR lowering achieved during right VNS [13, 14].

During the same period, we also provided the first evidence of the protective and only partially HR-dependent effect of VNS on reperfusion arrhythmias [15]. The mechanism underlying this effect was only recently unraveled, together with the understanding that reperfusion arrhythmias share common pathways with ischemia/reperfusion injury. In both settings, VNS exerts its protective effects [16–18] by complex intracellular pathways including activation, through Gi-coupled muscarinic receptors, of phosphatidylinositol 3‑kinase and Akt. These are the same pathways implicated in the protective effect of ischemic preconditioning [19]. Finally, VNS leads to the upregulation of the anti-apoptotic protein BCL‑2 and to the suppression of caspase‑3 in cardiomyocytes, thus protecting from cell death [20]. Notably, an increase in cardiac efferent vagal tone is also likely to mediate the beneficial effect of remote preconditioning [21, 22].

Studies of VNS on inflammation also produced interesting findings. In 2002, Tracy [23] provided conclusive evidence supporting the so-called cholinergic anti-inflammatory pathway, a neural mechanism that inhibits pro-inflammatory cytokine release via signals that require α7 cholinergic receptor expression on macrophages and other immunocompetent cells. In 2011 Calvillo et al. [24] demonstrated the cardiac effects of this pathway, underlying the crucial role played by nicotinic receptors in this HR-independent anti-inflammatory and anti-apoptotic effect of VNS, which produced a marked infarct size reduction following ischemia/reperfusion in rats.

Finally, the results of the first experimental study showing the benefit of chronic VNS for the treatment of HF were published in 2004 [25]. At 14 days after a large anterior myocardial infarction (MI) leading to HFrEF, rats were randomized to sham stimulation or active VNS (10 s on, 50 s off), at 20 Hz, with 0.2-ms pulses. The intensity of VNS was adjusted to reduce HR by 20 to 30 bpm from a starting value of 360 bpm. After 6 weeks of VNS, a duration mostly determined by the generator’s life, the treated animals showed a significantly better left ventricular (LV) function, a lower normalized biventricular weight, lower norepinephrine and B‑type natriuretic peptide (BNP) levels, and a strikingly better survival (86% vs. 50%) at 140 days compared to sham-operated animals, despite a similar infarct size. Notably, the lack of difference in the infarct size was expected due to the relatively long time elapsed between coronary artery ligation and the beginning of VNS.

Subsequently, between 2005 and 2013, the group of Hani Sabbah conducted several studies assessing the effects of right VNS on a canine model of chronic HF induced by coronary microembolizations [26, 27]. In the first two studies [26], the authors used the CardioFit system (see below) to deliver VNS. This system includes an intracardiac sensing lead aimed to synchronize VNS to the cardiac cycle and to continuously modulate VNS intensity based on the extent of acute HR reduction achieved during stimulation, which was set at 10% of the baseline HR. In the first study, 3 months of VNS, compared with sham operation, improved LV hemodynamics, decreased tumor necrosis factor (TNF)-α and interleukin (IL)-6 levels, tended to normalize nitric oxide synthase (NOS) expression, and dramatically increased the expression of Cx43, a gap-junction-forming protein whose reduction is associated with conduction blocks and increased arrhythmic susceptibility. Notably, the histological comparison between the right and the left VN performed at the end of the study revealed normal right VN fascicular and cellular structure. In the second study, the beneficial effects of a 3-month combination therapy of VNS and metoprolol on LV hemodynamics were proven to be additional to those achieved with metoprolol alone. Finally, in the third study [27], the group used a different VNS system (Boston Scientific Corporation) specifically set with a low stimulation intensity, unable to affect HR. Nonetheless, the beneficial effects of VNS on LV function were still evident, together with an improvement in NT-proBNP, pro-ANP, TNF‑α, IL‑6, BCL‑2, caspase‑3, Cx43, and the three isoforms of NOS.

In 2009, Zhang et al. [28] studied the effects of chronic VNS in a different model of HF, induced by high-rate ventricular pacing. Dogs in the active group received 8 weeks of high-rate ventricular pacing with concomitant VNS (20 Hz, pulse width 0.5 ms, duty cycle 14 s on and 12 s off), while the sham group only received pacing. The intensity of VNS was individually set before the start of pacing to reduce sinus rate by approximately 20%. Again, although HR was kept constant by pacing, VNS elicited significant benefits in LV hemodynamics, C‑reactive protein, norepinephrine and angiotensin II levels, HR variability, and baroreflex sensitivity compared to controls.

Notably, despite several demonstrations of the beneficial effects of VNS in post-MI animal models, data on timing, spatial distribution, and functional consequences of parasympathetic remodeling occurring after MI have long been lacking. Very recently, Vaseghi et al. [29] demonstrated that, as opposed to norepinephrine levels, cardiac acetylcholine levels are preserved 6–8 weeks after MI in border zones and in viable myocardium of infarcted hearts, supporting the anatomical integrity of the cardiac parasympathetic neuronal network. Yet, in vivo neuronal recordings from postganglionic parasympathetic neurons unraveled both abnormal firing frequency at rest and abnormal responses to stimuli, proving a profound disruption of parasympathetic cardiac control after MI and reinforcing the strong rationale for therapeutic interventions aimed at restoring a proper cardiac vagal output, such as VNS [30].

## Clinical experience

Chronic electrical unilateral (left-sided) VNS has been used clinically for decades for the management of drug-refractory epilepsy [31] and, more recently, also for resistant depression [32] with more than 100,000 patients treated all over the world and no major safety concerns.

Table 1 lists the five clinical studies of cervical VNS in HFrEF (four published, one ongoing), showing their main inclusion/exclusion criteria, the objectives, and the stimulation protocols. Table 2 presents the patient characteristics and 6‑month results of the four published studies.

**Table 1:** VNS studies: trial characteristics and stimulation protocols (modified from [4])

Parameter	CARDIOFIT (2011)	ANTHEM-HF (2014)	NECTAR-HF (2014)	INOVATE-HF (2016)	ANTHEM-HFrEF pivotal study (ongoing)
*Trial characteristics*					
Phase	I to II	I to II	II	III	III
Stimulation side	R	R vs. L	R	R	R
Control group	None	R vs. L	Stimulation off	GDMT	GDMT
Primary endpoint	Safety	LVESV; LVESD, LVEF	LVESD	Composite of death and HF hospitalization	Composite of cardiovascular death, or first HF hospitalization
Exclusion criteria for diabetic patients	Insulin-dependent diabetes mellitus or diabetic neuropathy	Autonomic or sensory neuropathy of any cause; HbA1C > 8% in the past 60 days	Type I diabetes, type II diabetes for over 5 years	Not specified	Not specified
NYHA Class	II–IV	II–III	II–III	III	III stable–II unstable
LVEF, LVEDD	≤ 35%	≤ 40%, LVEDD 50–80 mm	≤ 35%, LVEDD > 55 mm	≤ 40%, LVEDD 50–80 mm	≤ 35%, LVEDD < 80 mm
Rhythm, QRS duration (ms)	SR, QRS NA	SR, QRS ≤ 150	SR, QRS < 130	SR, QRS NA	Both SR an AF, QRS not specified
NT-pro BNP levels (pg/ml)	Not specified	Not specified	Not specified	Not specified	≥ 800 for patients in SR
					≥ 1200 for patients in AF
6MWT (m)	> 300	150–425	Not specified	Not specified	150–450
*Stimulation protocols*					
Implantable pulse generator	Cardiofit, BioControl Medical	Cyberonic IPG: Model 103	Precision, Boston Scientific	Cardiofit, BioControl Medical	VITARIA System (LivaNova)
Electrode lead	Asymmetric bipolar multi-contact cuff	Helical bipolar	Helical bipolar	Asymmetric bipolar multi-contact cuff	Helical bipolar
Asymmetric stimulation	Yes (afferent block above 4 mA)	No	No	Yes (afferent block above 4 mA)	No
ECG Synch	Yes	No	No	Yes	No
Current amplitude (mA)	4.1 ± 1.2 (range 1.1–5.5)	2.0 ± 0.6 (maximum 3)	1.4 ± 0.8 (range 0.3–3.5)	3.9 ± 1.0 at 6 months	NA
				Target 3.5–5.5	
Frequency (Hz)	1–3	10	20	1–2	NA
Duty cycle (%)	21	17.5	17	≤ 25%	NA
On/Off (s)	Variable	14/66	10/50	Variable	NA

*6MWT *6‑Minute Walking Test,* ANTHEM-HF* Autonomic Regulation Therapy via Left or Right Cervical Vagus Nerve Stimulation in Patients With Chronic Heart Failure, *GDMT* guideline-directed medical treatment, *HF* heart failure, *INOVATE-HF* Increase of Vagal Tone in Heart Failure, *L* left, *LVEDD* left ventricular end-diastolic diameter, *LVEF* left ventricular ejection fraction, *LVESD* left ventricular end-systolic diameter, *LVESV* left ventricular end-systolic volume, *NA* not available, *NECTAR-HF* Neural Cardiac Therapy for Heart Failure, *NYHA* New York Heart Association class, *R* right, *S* sinus rhythm., *Synch* synchronization

**Table 2:** VNS studies: baseline characteristics and 6 months results (modified from [5])

Parameter	CARDIOFIT (2011)	ANTHEM-HF (2014)	NECTAR-HF (2014)	INOVATE-HF (2016)
*Patient characteristics*				
Patients, *n* (male, %)	32 (30, 94)	60 (52, 87)	96, 87 paired	707 (558, 79)
Age (years)	56 ± 11	51 ± 12	59 ± 11	61 ± 10
Type II diabetes, %	NA	NA	26	36
NYHA II/III/IV	15/15/2	34/26/0	14/73/0	0/707/0
Ischemic HF, %	62	75	67	60
LVEF (%)	23 ± 8	32 ± 7	30 ± 6	25 ± 7
Basal LVESV (ml)	185 ± 63	108 ± 40	155 ± 58	103 ± 41 ml/sqm
HR (bpm)	82 ± 13	78 ± 10	69 ± 13	72 ± 12
ICD/CRT/none (%)	19/0/13	0/0/60	73/9/13	48/34/28
NT proBNP (pg/ml)	1316 (227–1997)	868 (322–1875)	879 (370–1843)	NA
hsCRP (mg/dl)	NA	1.7 (0.9–6.0)	0.18 (0.10–0.36), *n* = 96	NA
BB (%)	97	100	94	94
ACEi/ARB (%)	97	85	ACEi 78, ARB 25	89
MRA (%)	97	75	70	58
*6‑month results*				
Δ Mean HR at Holter (bpm)	0	−3.9	+0.5	NA
Δ LVEF (%)	+6.4	+4.5	+0.9	0
Δ LVESV (ml)	−25	−4.1	0	−3.7 ± 5.9 ml/sqm
Δ QoL	−17 (MLHF)	−18 (MLHF)	−8 (MLHF)	+5 (KCCQ)
NYHA improvement (%)	59	77	17	13
Δ 6MWT (m)	+60	+56	pVO_2_ + 0.7	+33
Δ NT proBNP (pg/ml)	−594 (*p* = 0.06)	+140	+93	NA

*6MWT *6‑Minute Walking Test,* ACEi* angiotensin-converting-enzyme inhibitor, *ARB* angiotensin II receptor blockers, *BB* beta-blocker, *CRP* C-reactive protein, *CRT* cardiac resynchronization therapy, *HF* heart failure, *ICD* implantable cardioverter defibrillator, *KCCQ* Kansas City Cardiomyopathy Questionnaire,* LVEF* left ventricular ejection fraction, *LVESV* left ventricular end-systolic volume, *MLHF* Minnesota Living with Heart Failure, *MRA* mineralocorticoid receptor antagonist, *NA* not available, *NYHA* New York Heart Association class, *NT proBNP* N-terminal pro B‑type natriuretic peptide, *pVO*_*2*_ peak oxygen consumption at cardiopulmonary exercise testing

The first proof-of-concept study of electrical VNS in HFrEF was performed as a single-center experience in Italy, with eight participants (all males: [33]). Based on the favorable preliminary results at 6 months, the study was subsequently extended to a multicenter single-arm open-label phase-II European study including 32 patients and using the same device (CardioFit 5000; [34]). The study, usually referred to as European Multicenter CardioFit Study, aimed to assess the safety and tolerability of VNS (primary endpoints) as well as to collect detailed efficacy data (secondary endpoints). The device had already been favorably tested in the first animal studies by Sabbah’s group and consisted of an implantable neurostimulator and two leads: one stimulating the right cervical VN, the other connected to a standard bipolar sensing electrode to sense each QRS complex in the right ventricle. The vagal electrode had been specifically designed to achieve a preferential, albeit not exclusive, stimulation of efferent fibers, through simultaneous cathodic stimulation and asymmetrical anodal block combined to a multi-contact cuff design (to maximize, at relatively high-current amplitudes, B‑type fiber recruitment while minimizing A‑type fiber recruitment). The implanted system provided a pulse synchronous (1–3 pulses per cardiac cycle) VNS at 1–3 Hz, with 10 s on and 30 s off, and was programmed to temporarily stop in the case of HR lowering below a predefined safety value, which was initially set at 55 bpm, thus forming a so-called closed-loop system. In five to six visits, VNS intensity was up-titrated to reach a mean level of 4.1 ± 1.2 mA; hoarseness and jaw pain were the main limiting symptoms to a further increase. Overall, the mean HR reduction achieved during the on-phase was modest (1.5 bpm as measured by a 24‑h Holter recording) but consistent across patients, with a small minority of patients showing as much as a 10-bpm acute decrease. At 6 months, ECG resting HR decreased from 82 ± 13 to 76 ± 13 bpm. Notably, HRV tended to increase during the study and the change in pNN50 was statistically significant, despite no changes in the mean HR on Holter ECG. The latter finding was thought to reflect mainly a greater level of physical activity due to the improved HF status. Patients were in a very advanced baseline condition and on optimized and stable medical therapy including beta-blockers, angiotensin-converting enzyme inhibitors/angiotensin receptor blockers, and loop diuretics. Still, after 6 months of VNS, quality of life score (QoL) and functional capacity based on NYHA class and 6‑minute walk test (6MWT) significantly improved (see Table 2), together with a significant improvement in LV end-systolic volume (LVESV) and left ventricular ejection fraction (LVEF, from 22 ± 7% to 29 ± 8%) as assessed by blinded ultrasound analysis. The results were maintained in those who reached the 1‑ and 2‑year follow-up, with no major safety concerns. Notably, 59% of patients had an implantable cardioverter defibrillator (ICD) at baseline, but none had cardiac resynchronization therapy (CRT).

The second study assessing the effects of VNS in HFrEF was the Autonomic Regulation Therapy for the Improvement of Left Ventricular Function and Heart Failure Symptoms (ANTHEM-HF) study [35]. The ANTHEM-HF was a multicenter, open-label, phase II clinical trial enrolling patients at 10 Indian sites. Overall, 60 patients (NYHA class II–III, LVEF ≤ 40%, left ventricular end diastolic diameter [LVEDD], between 50 and 80 mm, QRS < 150 ms, none with ICD or CRT) were randomized to receive either left (*n* = 31) or right (*n* = 29) VNS system implantation (Demipulse Model 103 pulse generator and PerenniaFLEX Model 304 lead, Cyberonics, Houston, TX, USA). The stimulation system had already been approved for clinical use for drug-refractory epilepsy and tested in the high-rate pacing-induced canine model of HF. Vagal nerve stimulation was performed through an open-loop system lacking ECG synchronization capability and delivering a 14‑s on and 66‑s off stimulation, at 10 Hz; the vagal electrode was not designed for asymmetrical stimulation. After a 10-week up-titration period, a mean output current of 2.0 ± 0.6 mA was reached, with a good safety profile. The pooled right and left efficacy analysis at 6 months showed a significant (+4.5% in absolute values) increase in LVEF and a nonsignificant decrease of LVESV (co-primary endpoints). Further, QoL and NYHA also significantly improved. Right VNS was associated with a trend toward a greater benefit compared to left-sided stimulation. Improvements in cardiac function and HF symptoms seen after 6 months of VNS were maintained at 12 months [36], and until 42 months [37], with no significant difference between right- and left-sided VNS. A recent subanalysis including a 24- and a 36-month follow-up, confirmed the long-lasting, beneficial effect of VNS on autonomic tone (HRV), baroreceptor sensitivity (HR turbulence), and cardiac electrical stability as assessed by T‑wave alternans, R‑wave and T‑wave heterogeneity [38]. Additionally, a significant reduction of not-sustained ventricular tachycardia episodes at 12, 24, and 36 months was also reported [38].

The Neuronal Cardiac Therapy for Heart Failure (NECTAR-HF) study was a phase II, multicenter, sham-controlled study including 96 patients with NYHA class II–III, LVEF < 35%, a QRS < 130 ms and LVEDD > 55 mm [39]. All patients underwent implantation and were then randomized 2:1 to active VNS or sham treatment for the first 6 months; subsequently, VNS was turned on in all patients. The majority had an ICD, only few had CRT. The primary safety endpoint was 18-month all-cause mortality. The VNS system, sponsored by Boston Scientific (MN, USA), consisted of a rechargeable device already approved for chronic pain therapy (Precision) and an investigational helical bipolar vagal electrode not pursuing preferential stimulation and relatively similar to the one used in ANTHEM. After a 4-week up-titration period, a mean stimulation intensity amplitude of 1.4 ± 0.8 mA was reached at a stimulation frequency of 20 Hz. At 6‑month follow-up, left ventricular end systolic diameter (LVESD, primary efficacy endpoint) was unchanged, as was LVEDD, LVESV, LVEF, peak VO_2_ at cardiopulmonary exercise test, and NT-proBNP (all additional secondary endpoints). Yet, there was a statistical improvement in QoL score and in NYHA class in treated patients. The 18-month results (with all patients on active VNS) showed the persistence of a favorable adverse event profile with acceptable survival rate (95%). However, LVESD and LVEF did not change in the crossover group, confirming the 6‑month findings. Finally, QoL scores did not change either, while NYHA class improved in all patients over the 18-month study period. Additionally, a new technique to detect subtle HR changes, namely, tridimensional heat maps, was applied to 24‑h Holter ECG obtained at 6 and at 12 months: only 12% of the studies showed VNS-evoked HR responses, as opposed to zero studies in the sham arm [40]. This rare occurrence of even minimal HR effects during vagal stimulation is at variance with the findings with the same device in the experimental studies and may suggest lower amplitudes of stimulation and lower fiber recruitment as a reason for the different effects between the preclinical and the clinical settings. In the NECTAR-HF trial, no apparent differences were found in conventional measures of frequency and time domain HR variability when comparing treated patients with and without evidence of VNS-evoked HR changes in the heat map analysis.

The last and largest clinical trial of VNS in HFrEF completed so far is the Increase of Vagal Tone in Heart Failure (INOVATE-HF), a phase III international, multicenter, randomized trial aimed at assessing the efficacy and safety of chronic right-sided VNS using the CardioFit system [41]. Beginning in 2011, 707 patients with NYHA class III, LVEF ≤ 40%, and LVEDD between 50 and 80 mm were enrolled at 85 centers in the United States, Europe, and Israel and randomized in a 3:2 ratio to either active treatment (implanted) or continuation of medical therapy (not implanted). Except for a slightly lower LVEF in the VNS group, the remaining baseline characteristics were well balanced. Most patients had cardiac devices, including one-third with CRT. The primary efficacy endpoint was the composite of all-cause mortality or unplanned HF hospitalization equivalent, using a time-to-first-event analysis. Freedom from procedure and system-related complication events at 90 days and number of patients with all-cause death or complications at 12 months were the two co-primary safety endpoints. The study was stopped for futility in December 2015 after the second planned interim analysis. The mean follow-up period was 16 months (range: 0.1–52) and the mean stimulation current was 3.9 ± 1.0 mA, with 73% of patients achieving the goal of ≥ 3.5 mA. The LVESV index, a pre-specified secondary endpoint, was unchanged, whereas QoL, NYHA class, and 6 MWT distance were improved by VNS (*p* < 0.05), although the unblinded nature of the study should be kept in mind. Subgroup analysis failed to detect any significant interaction between the primary endpoint and age, 6MWT at baseline, HF etiology, diabetes, and CRT.

## Open issues and expectations from the ANTHEM-HFrEF pilot study

The strong pathophysiological rationale, combined with the favorable results of preclinical studies and of the first two, not controlled, clinical studies set great expectations for VNS in HFrEF, which were met by disappointment due to the neutral results of NECTAR-HF and of INOVATE-HF, leading to a general loss of confidence in the technique. It should be considered, however, that the trials were undertaken in the absence of adequate knowledge on the dose–response relationship of VNS, on the subgroups of patients most likely to benefit from VNS, and on the most suitable study design.

### The dose–response issue

The concept of “dose” for electrical therapies is considerably more complex than for pharmacological therapies, since more than 10 different parameters can be modulated at the same time, with hundreds of possible combinations. Nonetheless, clinical trials were started long before the dose issue was solved, resulting in different and largely not comparable VNS protocols. For instance, the mean current outputs attained in NECTAR-HF (1.3 mA at 20 Hz) and ANTHEM-HF (2.0 mA at 10 Hz) were markedly lower than those achieved in both the CardioFit pilot trial (4.2 mA, at 1–3 Hz) and the INOVATE-HF (3.9 at 1–3 Hz). Importantly, in the setting of VNS for HF, despite a slow and gradual up-titration process, the maximum current amplitude that can be reached is generally limited by off-target side effects such as local pain, voice alteration, and coughing, reflecting the lower threshold for recruitment of A‑type fibers of the recurrent laryngeal nerve compared to cardiac B‑ and C-type fibers. In vivo, the maximum tolerated current is inversely related to pulse frequency, thus explaining the low intensity reached in the NECTAR-HF study. Nonetheless, both the ANTHEM-HF and the CardioFit trial, despite quite different stimulation protocols, achieved good efficacy results in association with proofs of acute and chronic VN engagement as assessed by traditional [34, 35] and advanced HR dynamics [42, 43]. On the other hand, the INOVATE-HF failed in improving clinical outcomes, thus reducing the likelihood that an inadequate efferent fiber recruitment caused by insufficient stimulation amplitude could be the main explanation for the divergent results. Unfortunately, data on HR dynamics in the treatment group of the INOVATE-HF trial have not been reported yet, and therefore the amount of vagal engagement cannot be estimated. In addition to stimulus amplitude, the choice of the duty cycle, too, was empiric and mainly driven by the need to minimize side effects. Notably, the duty cycle in the INOVATE-HF and CardioFit pilot trials was variable and not standardized.

In recent years, some attempts to shed light on the complex dose issues of VNS in HF in tailored preclinical studies were reported. In 2013, Kong et al. [44] evaluated the effect of different right cervical VNS protocols in reducing the infarct size in anesthetized rats with acute MI, finding an interesting disconnection between the combination of parameters that was most effective in reducing infarct size (lower amplitude, lower frequency, 2 Hz, and longer duration of stimulation) and that which produced the greater HR decrease. These data support the idea that a purely efferent VNS, even if feasible, might not be conceptually ideal since afferent fiber activation may provide a significant contribution to the beneficial effects of VNS. Indeed, back in 1973 [45] an elegant study on anesthetized cats by Schwartz et al. demonstrated that afferent VNS at the cervical level (performed on the cut central end of the nerve, to completely avoid efferent stimulation) reduces ipsi- and contralateral sympathetic efferent output at the cardiac level, while increasing contralateral cardiac vagal output as assessed via single-fiber recording. The relative contribution of afferent versus efferent VN activation to the acute HR responses elicited during the active phase of chronic right VNS was recently evaluated in a canine conscious model [46]. A wide range of stimulation parameters were used to define optimal protocols for bidirectional bioelectronic cardiac control, including electrode configuration (anode/cathode inversion), pulse frequency (2–20 Hz), intensity (0–3.5 mA), and pulse widths (130–750 μs). The HR responses were determined for each combination over 14 months. At low intensities and higher-frequency VNS, HR increased during the VNS active phase due to afferent modulation of parasympathetic central drive. At higher intensity, afferent and efferent fiber activation were balanced, and a null HR response was evoked. Finally, as intensity further increased, a reduction in HR was observed during the active phase of VNS. The operating point, based on frequency–amplitude–pulse width, where a null HR response was evoked during the on-phase of VNS, was defined as the neuronal fulcrum. The authors also demonstrated that chronic VNS, delivered within the constraints of the neuronal fulcrum, was able to maintain the circadian control of HRV.

The ANTHEM-HF trial was the only study delivering VNS according to the principium of the neural fulcrum at a pulse frequency (10 Hz) that is similar to the synaptic efficacy for intrinsic cardiac neurons [47].

### Patient selection and blinding issues

As for any therapeutic intervention, proper patient selection is crucial for enrolling the participants most likely to benefit from ART. For instance, patients with a more pronounced autonomic imbalance are expected to show a better response to ART, thus the development of a suitable screening tool to assess sympathovagal balance should be pursued. Relatively easy to obtain, direct and indirect autonomic measurements such as baseline levels of serum catecholamines and a standardized way to assess HR dynamics though Holter ECG should be implemented both as inclusion criteria and as a tool to detect a proper vagal engagement, with the potential addition of more sophisticated measures such as spontaneous BRS, or muscle sympathetic nerve activity. For instance, baseline HR was appreciably higher in the first two, successful studies (82 ± 13 bpm in the Cardiofit study, 78 ± 10 bpm in ANTHEM-HF), as compared to the last two studies (70 ± 13 bpm in NECTAR-HF 69 ± 13 bpm INOVATE-HF), suggesting a more pronounced sympathetic overactivity.

Due to the strong anti-inflammatory effect of VNS, different baseline levels of inflammation across studies might also have influenced the observed results. Baseline levels of high-sensitivity (hs) TNF‑α, hs IL‑6, and hsCRP in NECTAR-HF were lower than values found in most studies with HF (including the ANTHEM-HF, the only other study of VNS in HFrEF that provided baseline CRP levels) and more similar to those of healthy individuals [48], suggesting that patients enrolled in NECTAR-HF had little active inflammation and therefore less chance of benefiting from the anti-inflammatory effect of VNS [23]. Yet, it must be acknowledged that a recent subanalysis of the ANTHEM-HF study demonstrated that overall symptomatic and functional improvement during chronic VNS was independent of baseline NTproBNP levels [49]. In turn, some data suggest that inflammation increases NT-proBNP levels more than BNP levels, particularly in the setting of HFrEF [50].

Diabetic patients and non-responders to CRT might also be less likely to respond to VNS, albeit no significant interaction was found in the INOVATE-HF trial. The former may suffer from latent diabetic neuropathy that typically affects the long parasympathetic fibers first; the latter have a high chance of being poor responders to any therapeutic intervention due to several factors, including a large LV scar. Accordingly, a post hoc exploratory analysis of INOVATE-HF including patients with no CRT, a QRS duration < 130 ms, and a baseline ability to walk of more than 300 m (the inclusion criteria used in CardioFit) showed a weak favorable trend versus reverse LV remodeling.

Finally, another methodological issue for ART is blinding. On one hand, conducting un-blinded clinical trials may lead to bias and question the study results, on the other hand, achieving proper blinding is a significant challenge in device-based trials. For instance, the 6‑month blinding assessment of the NECTAR-HF showed that 70% of the patients assigned to active VNS properly guessed their randomization group, probably due to the perception of ongoing stimulation or to side effects.

### ANTHEM-HFrEF study

An adaptive, open-label, randomized, controlled pivotal study (ANTHEM-HFrEF) is currently undergoing to further evaluate VNS in patients with advanced HF [51]. The ANTHEM-HFrEF is randomizing patients (2:1) to VNS plus guideline-directed medical therapy (GDMT) or GDMT alone. The estimated study completion date is December 2024. In the trial, VNS is delivered using the VITARIA System (LivaNova) and according to the principle of the neuronal fulcrum. ANTHEM-HFrEF utilizes an innovative adaptive design as allowed by the new Food and Drug Administration breakthrough devices program. Indeed, the primary outcome will be a composite of cardiovascular death, or first HF hospitalization traditionally assessed, but the sample size will be determined using a Bayesian adaptive approach.

## Conclusion

In conclusion, although a very strong pathophysiological rationale, combined with highly convincing preclinical and promising pilot studies, had set great hope for vagal nerve stimulation, the two large, controlled studies performed to date have failed to fulfill the expectations. Yet, it must be acknowledged that clinical translation was performed before a sufficient degree of understanding of the complex pathophysiology of autonomic electrical modulation had been achieved, therefore jeopardizing its potential benefit. An additional randomized study is currently underway using an innovative adaptive design and a refined approach in an attempt to solve the dose issues.

## Declarations

For this article no studies with human participants or animals were performed by any of the authors. All studies performed were in accordance with the ethical standards indicated in each case.

## References

[1] La Rovere MT, Bigger JT, Marcus FI (1998). Baroreflex sensitivity and heart-rate variability in prediction of total cardiac mortality after myocardial infarction. ATRAMI (Autonomic Tone and Reflexes After Myocardial Infarction) Investigators. Lancet.

[2] La Rovere MT, Pinna GD, Maestri R (2009). Prognostic implications of baroreflex sensitivity in heart failure patients in the beta-blocking era. J Am Coll Cardiol.

[3] Adamson PB, Smith AL, Abraham WT (2004). Continuous autonomic assessment in patients with symptomatic heart failure: prognostic value of heart rate variability measured by an implanted cardiac resynchronization device. Circulation.

[4] De Ferrari GM (2014). Vagal stimulation in heart failure. J Cardiovasc Transl Res.

[5] De Ferrari GM, Dusi V (2015). Stimolazione vagale nel trattamento dello scompenso cardiaco [Vagus nerve stimulation for the treatment of heart failure]. G Ital Cardiol.

[6] Armour JA, Murphy DA, Yuan BX (1997). Gross and microscopic anatomy of the human intrinsic cardiac nervous system. Anat Rec.

[7] Settell ML, Pelot NA, Knudsen BE (2020). Functional vagotopy in the cervical vagus nerve of the domestic pig: implications for the study of vagus nerve stimulation. J Neural Eng.

[8] Einbrodt P (1859). Ueber Herzreizung und ihr Verhaeltnis zum Blutdruck.

[9] Yoon MS, Han J, Tse WW (1977). Effects of vagal stimulation, atropine, and propranolol on fibrillation threshold of normal and ischemic ventricles. Am Heart J.

[10] Kolman BS, Verrier RL, Lown B (1975). The effect of vagus nerve stimulation upon vulnerability of the canine ventricle: role of the sympathetic-parasympathetic interactions. Circulation.

[11] Kent KM, Smith ER, Redwood DR (1973). Electrical stability of acutely ischemic myocardium: Influences of heart rate and vagal stimulation. Circulation.

[12] Myers RW, Pearlman AS, Hyman RM (1974). Beneficial effects of vagal stimulation and bradycardia during experimental acute myocardial ischemia. Circulation.

[13] Vanoli E, De Ferrari GM, Stramba-Badiale M (1991). Vagal stimulation and prevention of sudden death in conscious dogs with a healed myocardial infarction. Circ Res.

[14] Dusi V, De Ferrari GM, Schwartz PJ (2020). There are 100 ways by which the sympathetic nervous system can trigger life-threatening arrhythmias. Eur Heart J.

[15] Zuanetti G, De Ferrari GM, Priori SG (1987). Protective effect of vagal stimulation on reperfusion arrhythmias in cats. Circ Res.

[16] Kakinuma Y, Ando M, Kuwabara M (2005). Acetylcholine from vagal stimulation protects cardiomyocytes against ischemia and hypoxia involving additive non-hypoxic induction of HIF-1alpha. FEBS Lett.

[17] Uemura K, Zheng C, Li M (2010). Early short-term vagal nerve stimulation attenuates cardiac remodeling after reperfused myocardial infarction. J Card Fail.

[18] Mioni C, Bazzani C, Giuliani D (2005). Activation of an efferent cholinergic pathway produces strong protection against myocardial ischemia/reperfusion injury in rats. Crit Care Med.

[19] Krieg T, Qin Q, Philipp S (2004). Acetylcholine and bradykinin trigger preconditioning in the heart through a pathway that includes Akt and NOS. Am J Physiol Heart Circ Physiol.

[20] Katare RG, Ando M, Kakinuma Y (2009). Vagal nerve stimulation prevents reperfusion injury through inhibition of opening of mitochondrial permeability transition pore independent of the bradycardiac effect. J Thorac Cardiovasc Surg.

[21] Crimi G, Pica S, Raineri C (2013). Remote ischemic post-conditioning of the lower limb during primary percutaneous coronary intervention safely reduces enzymatic infarct size in anterior myocardial infarction: a randomized controlled trial. JACC Cardiovasc Interv.

[22] Donato M, Buchholz B, Rodríguez M (2013). Role of the parasympathetic nervous system in cardioprotection by remote hindlimb ischaemic preconditioning. Exp Physiol.

[23] Tracey K (2002). The inflammatory reflex. Nature.

[24] Calvillo L, Vanoli E, Andreoli E (2011). Vagal stimulation, through its nicotinic action, limits infarct size and the inflammatory response to myocardial ischemia and reperfusion. J Cardiovasc Pharmacol.

[25] Li M, Zheng C, Sato T (2004). Vagal nerve stimulation markedly improves long-term survival after chronic heart failure in rats. Circulation.

[26] Sabbah HN, Ilsar I, Zaretsky A (2011). Vagus nerve stimulation in experimental heart failure. Heart Fail Rev.

[27] Hamann JJ, Ruble SB, Stolen C (2013). Vagus nerve stimulation improves left ventricular function in a canine model of chronic heart failure. Eur J Heart Fail.

[28] Zhang Y, Popovic ZB, Bibevski S (2009). Chronic vagus nerve stimulation improves autonomic control and attenuates systemic inflammation and heart failure progression in a canine high-rate pacing model. Circ Heart Fail.

[29] Vaseghi M, Salavatian S, Rajendran PS (2017). Parasympathetic dysfunction and antiarrhythmic effect of vagal nerve stimulation following myocardial infarction. JCI Insight.

[30] Dusi V, De Ferrari GM, Mann DL (2020). Cardiac sympathetic-parasympathetic interaction: the endless story of yin and yang. JACC Basic Transl Sci.

[31] Uthman BM, Reichl AM, Dean JC (2004). Effectiveness of vagus nerve stimulation in epilepsy patients: a 12-year observation. Neurology.

[32] Shuchman M (2007). Approving the vagus-nerve stimulator for depression. N Engl J Med.

[33] Schwartz PJ, De Ferrari GM, Sanzo A (2008). Long term vagal stimulation in patients with advanced heart failure: first experience in man. Eur J Heart Fail.

[34] De Ferrari GM, Crijns HJ, Borggrefe M (2011). Chronic vagus nerve stimulation: a new and promising therapeutic approach for chronic heart failure. Eur Heart J.

[35] Premchand RK, Sharma K, Mittal S (2014). Autonomic regulation therapy via left or right cervical vagus nerve stimulation in patients with chronic heart failure: results of the ANTHEM-HF trial. J Card Fail.

[36] Premchand RK, Sharma K, Mittal S (2016). Extended follow-up of patients with heart failure receiving autonomic regulation therapy in the ANTHEM-HF study. J Card Fail.

[37] Sharma K, Premchand RK, Mittal S (2021). Long-term follow-up of patients with heart failure and reduced ejection fraction receiving autonomic regulation therapy in the ANTHEM-HF pilot study. Int J Cardiol.

[38] Nearing BD, Anand IS, Libbus I (2021). Vagus nerve stimulation provides multiyear improvements in autonomic function and cardiac electrical stability in the ANTHEM-HF study. J Card Fail.

[39] Zannad F, De Ferrari GM, Tuinenburg AE (2015). Chronic vagal stimulation for the treatment of low ejection fraction heart failure: results of the NEural Cardiac TherApy foR Heart Failure (NECTAR-HF) randomized controlled trial. Eur Heart J.

[40] De Ferrari GM, Stolen C, Tuinenburg AE (2017). Long-term vagal stimulation for heart failure: eighteen month results from the NEural Cardiac TherApy foR Heart Failure (NECTAR-HF) trial. Int J Cardiol.

[41] Gold MR, Van Veldhuisen DJ, Hauptman PJ (2016). Vagus nerve stimulation for the treatment of heart failure: the INOVATE-HF trial. J Am Coll Cardiol.

[42] Nearing BD, Libbus I, Amurthur B (2016). Acute autonomic engagement assessed by heart rate dynamics during vagus nerve stimulation in patients with heart failure in the ANTHEM-HF trial. J Cardiovasc Electrophysiol.

[43] Libbus I, Nearing BD, Amurthur B (2017). Quantitative evaluation of heartbeat interval time series using Poincaré analysis reveals distinct patterns of heart rate dynamics during cycles of vagus nerve stimulation in patients with heart failure. J Electrocardiol.

[44] Kong SS, Liu JJ, Hwang TC (2012). Optimizing the parameters of vagus nerve stimulation by uniform design in rats with acute myocardial infarction. PLoS ONE.

[45] Schwartz PJ, Pagani M, Lombardi F (1973). A cardiocardiac sympathovagal reflex in the cat. Circ Res.

[46] Ardell JL, Nier H, Hammer M (2017). Defining the neural fulcrum for chronic vagus nerve stimulation: implications for integrated cardiac control. J Physiol.

[47] Hardwick JC, Southerland EM, Ardell JL (2008). Chronic myocardial infarction induces phenotypic and functional remodeling in the guinea pig cardiac plexus. Am J Physiol Regul Integr Comp Physiol.

[48] Testa M, Yeh M, Lee P (1996). Circulating levels of cytokines and their endogenous modulators in patients with mild to severe congestive heart failure due to coronary artery disease or hypertension. J Am Coll Cardiol.

[49] Anand I, Ardell JL, Gregory D (2020). Baseline NT-proBNP and responsiveness to autonomic regulation therapy in patients with heart failure and reduced ejection fraction. Int J Cardiol Heart Vasc.

[50] Jensen J, Ma LP, Fu ML (2010). Inflammation increases NT-proBNP and the NT-proBNP/BNP ratio. Clin Res Cardiol.

[51] Konstam MA, Udelson JE, Butler J (2019). Impact of autonomic regulation therapy in patients with heart failure: ANTHEM-HFrEF pivotal study design. Circ Heart Fail.

